# Context dependent interactions between protozoan infections and cancer biology-a retrospective review

**DOI:** 10.3389/fimmu.2026.1968151

**Published:** 2026-10-09

**Authors:** Fanglei Yu, Zheng Wu, Yaru Huang, Xiaochen Qi, Jun Wu, Yuanhua Qin

**Affiliations:** 1 The First Affiliated Hospital, Dalian Medical University, Dalian, China; 2 College of Basic Medical Science, Dalian Medical University, Dalian, China; 3 The Second Affiliated Hospital, Dalian Medical University, Dalian, China

**Keywords:** biotherapy, chronic infection, context-dependence, immune evasion, protozoa, tumor immunity, tumor microenvironment

## Abstract

Protozoan infections can influence tumor biology through immune modulation, effects on tumor cells and vasculature, and changes in mucosal homeostasis. The supporting evidence, however, differs substantially among organisms and experimental settings. Selected studies of Toxoplasma gondii, Plasmodium species, and Trypanosoma cruzi demonstrate tumor suppression in cell and animal models, including immune activation, altered myeloid-cell function, and inhibition of angiogenesis. Associations between intestinal or urogenital protozoa and human cancers are mainly observational and do not establish carcinogenesis. Malaria associated with Plasmodium falciparum in holoendemic areas provides a distinct cancer-associated context that cannot be equated with experimental antitumor interventions. This narrative review distinguishes population associations, mechanistic observations, causal evidence within experimental models, and human therapeutic investigation. It evaluates strain or subtype, infection burden and duration, tissue tropism, host immune status, and observation timing as candidate modifiers rather than universally validated determinants. Direct tumor-cell effects, vascular effects, and host-immune-mediated effects are considered separately. Particular attention is given to inconsistent Trichomonas vaginalis and Blastocystis findings, limitations of murine models, confounding and reverse causation, and the safety of live-organism approaches in immunocompromised patients. Registered Plasmodium-based oncology studies demonstrate clinical investigation, but registration does not establish therapeutic benefit. Most engineered parasites and parasite-derived platforms discussed remain preclinical. The proposed framework organizes heterogeneous evidence and identifies testable questions for safer translational research; it does not imply that natural protozoan infection provides a predictable clinical benefit.

## 1 Introduction

The tumor microenvironment comprises malignant cells, stromal cells, and infiltrating immune populations whose interactions influence tumor growth and treatment response (1). Dendritic-cell antigen presentation (2), natural killer (NK) and innate lymphoid cell (ILC) function (3), and myeloid-derived suppressor cell (MDSC) activity (4) are relevant components. Infectious agents can modify these processes, making pathogen-associated cancer biology an important area of investigation (5). Protozoa occupy diverse intracellular, tissue, and mucosal niches and employ distinct mechanisms of immune modulation; they should not be treated as a biologically uniform group.

Experimental observations linking protozoa to tumor suppression date to the 1940s. Roskin reported effects of protozoan infection on transplanted tumors (6), and Hauschka and Goodwin investigated a Trypanosoma cruzi preparation termed KR in malignant mouse tumors (7). These historical experiments provide context for modern research but do not establish efficacy in humans. Cancer-associated effects also occur in different exposure settings: malaria caused by P. falciparum in holoendemic areas is classified by IARC as probably carcinogenic to humans (Group 2A), with evidence relating to endemic Burkitt lymphoma (8). This exposure is distinct from rodent malaria models and from parasite-derived targeting molecules.

Molecular studies have identified protozoan effectors that alter host signaling, immune-cell function, and tissue responses. Whether such changes modify a tumor requires investigation in a defined cancer model. An immune-evasion mechanism demonstrated during infection cannot automatically be interpreted as either tumor-promoting or tumor-suppressive.

A productive comparison therefore begins with the biological unit being studied. Natural infection exposes multiple tissues to a changing organism burden and recruits both protective and regulatory responses. An intratumoral inoculation places an experimental preparation directly into an established lesion. A purified protein or an engineered antigen carrier isolates only part of the parasite’s biology. These exposures may share an organism of origin while differing in their cellular targets, distribution, and duration. Distinguishing them helps connect the historical infection experiments with contemporary molecular work without assuming that a successful product reproduces the overall consequences of infection. It also clarifies why an association with cancer occurrence and an intervention that slows an existing tumor need separate explanations. The central question is which defined host–parasite interaction changes a specified cancer endpoint, and under what conditions that effect can be reproduced.

A systematic review of experimental T. gondii studies described both tumor-suppressive and tumor-promoting findings across heterogeneous models (9). It used descriptive synthesis rather than quantitative meta-analysis. Variation in strain, parasite preparation, infection stage, and host background motivates a context-dependent interpretation, but differences between studies do not establish that each variable causes a reversal of tumor outcome.

This narrative review examines experimental studies, population observations, and translational investigations of protozoan–tumor interactions. Section 2 distinguishes infection-related mechanisms from mechanisms tested in cancer models. Sections 3 and 4 consider tumor-suppressive findings and cancer-associated effects, respectively. Section 5 evaluates candidate contextual modifiers, Section 6 addresses human epidemiology and opportunistic infection, and Section 7 separates preclinical platforms from registered clinical investigation. Limitations are discussed in Section 8 before the conclusions in Section 9. The evidence framework, research milestones, selected findings, and translational stages are summarized in **Figures 1**–**4**, respectively.

**Figure 1:**
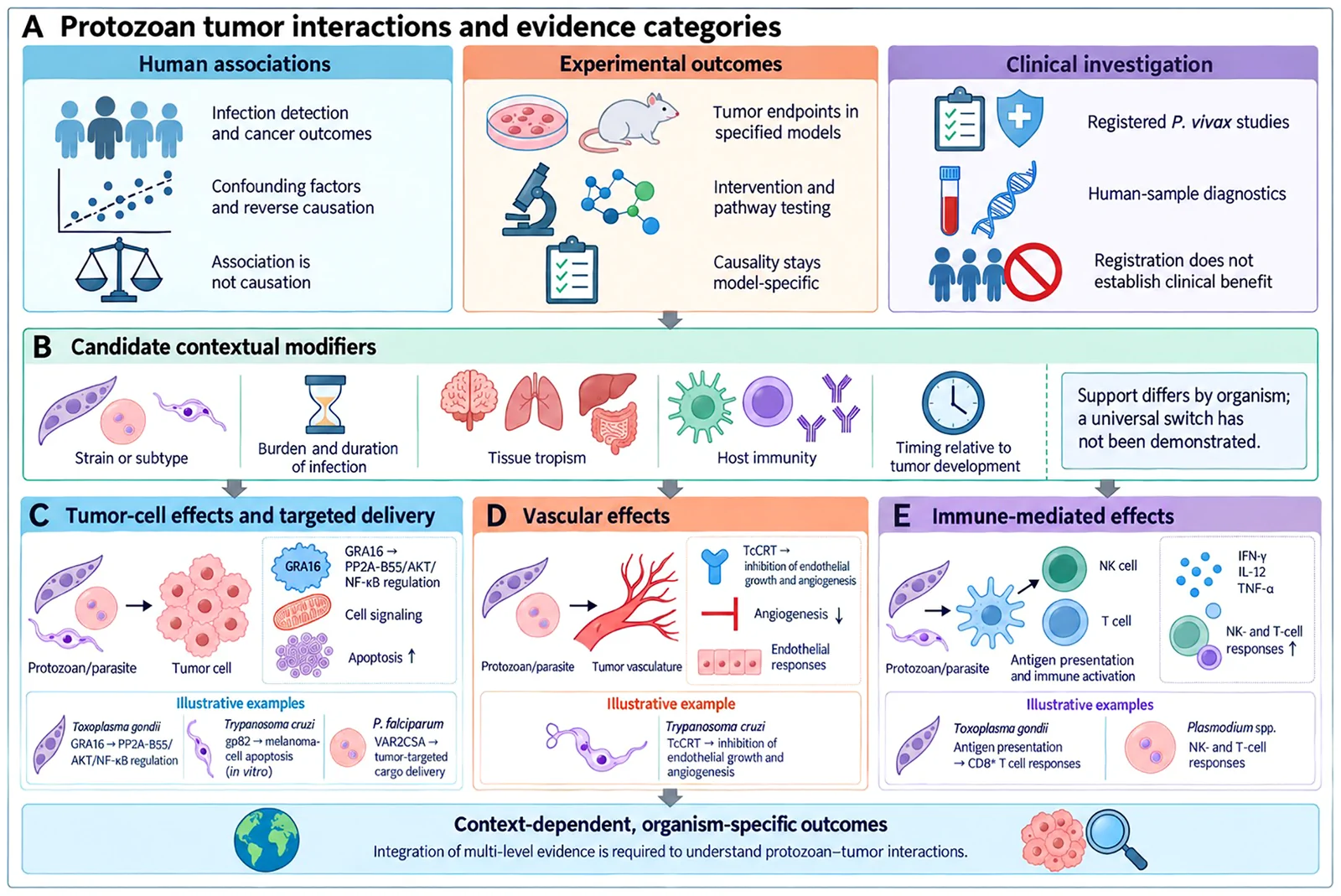
Evidence categories and candidate modifiers of protozoan–tumor interactions. Panel **(A)** distinguishes human associations, experimental outcomes, and clinical investigation. Observational associations require evaluation of confounding and reverse causation; experimental causality remains specific to the tested model; trial registration and human-sample diagnostics do not establish therapeutic benefit. Panel **(B)** presents strain or subtype, exposure burden and duration, tissue tropism, host immunity, and timing as candidate contextual modifiers. Their support differs among organisms, and a universal switch has not been demonstrated. Duration and timing are related but distinct exposure characteristics, as discussed in Section 5. Panels **(C–E)** distinguish direct tumor-cell, vascular, and immune-mediated mechanisms. Representative evidence includes GRA16-associated PP2A-B55/NF-κB regulation in lung carcinoma cells (52), (T) cruzi gp82-associated melanoma-cell apoptosis (53), TcCRT-associated inhibition of angiogenesis (54, 55), and host-immune-dependent tumor responses in selected Toxoplasma and Plasmodium models (56–59). Parasite illustrations indicate categories of interaction rather than demonstrating that each organism uses every displayed pathway. Cytokine modulation or immune-cell changes alone do not establish a tumor endpoint.

**Figure 2:**
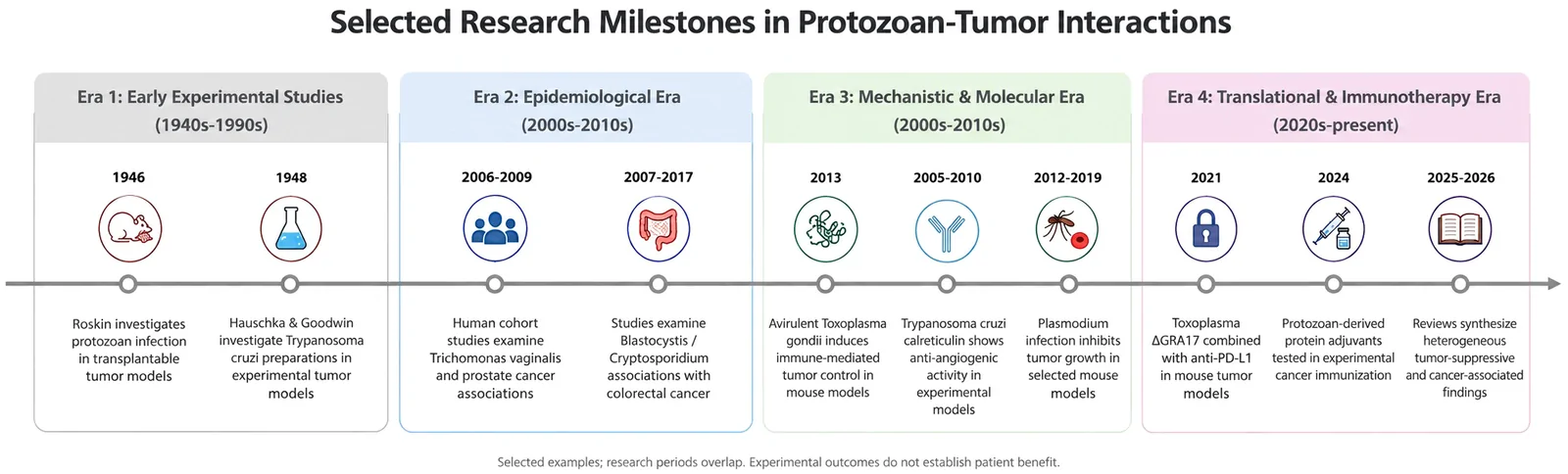
Selected research milestones in protozoan-tumor interactions. The timeline summarizes historical experimental investigations (6, 7), human association studies (101, 116, 117), mechanistic tumor-model work involving TcCRT and Toxoplasma (54–57), Plasmodium experiments (59, 71), and more recent checkpoint-combination and protein-adjuvant studies (36, 68). Research periods overlap; their placement does not imply increasing clinical efficacy. Reviews synthesize heterogeneous findings rather than validate a universal switch (9, 45–51).

**Figure 3:**
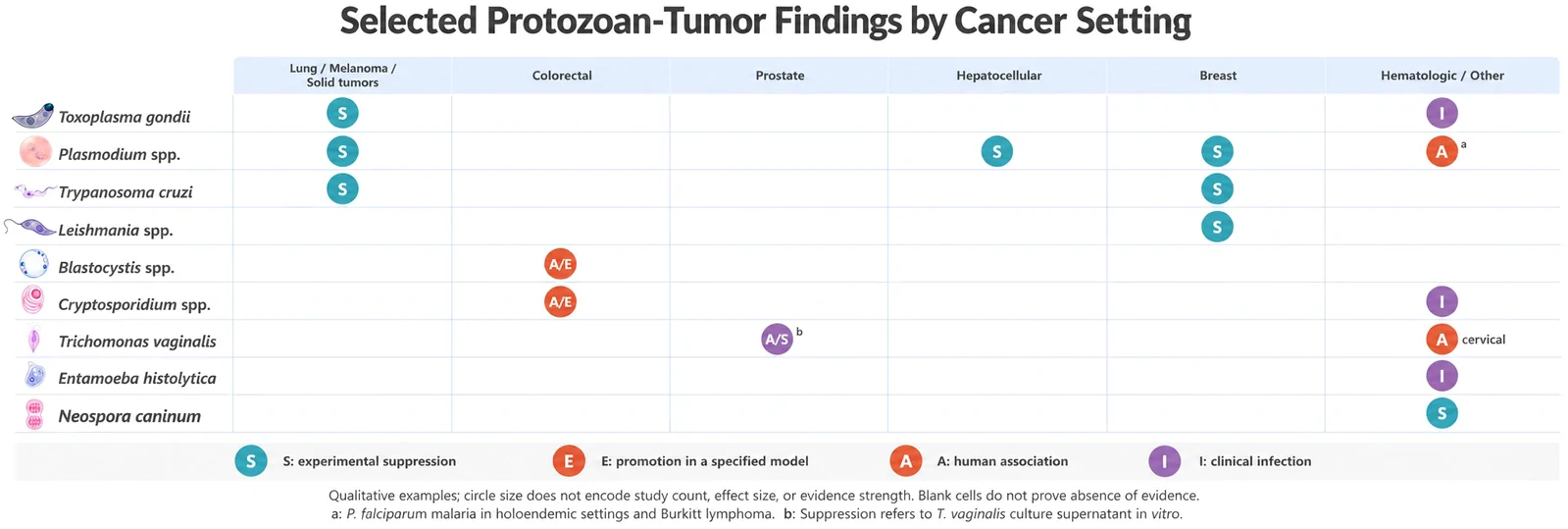
Selected protozoan-tumor findings by cancer setting. The matrix summarizes selected findings by species and cancer setting. Equal-sized markers indicate selected evidence categories: S, experimental suppression; E, promotion in a specified model; A, human association; and I, clinical infection. Combined labels denote different evidence types within a broad cancer setting, not a demonstrated reversal within one system. Marker size does not encode study count, effect size, or evidence strength; blank cells do not prove absence of evidence. Human associations and infection observations are not proof of cancer causation. P. falciparum malaria in holoendemic settings and Burkitt lymphoma (8) are distinguished from experimental Plasmodium tumor suppression. The T. vaginalis suppression label refers to culture-supernatant experiments *in vitro* (114). **Tables 1**-**3** and Sections 3–6 provide the underlying sources and limitations.

**Figure 4:**
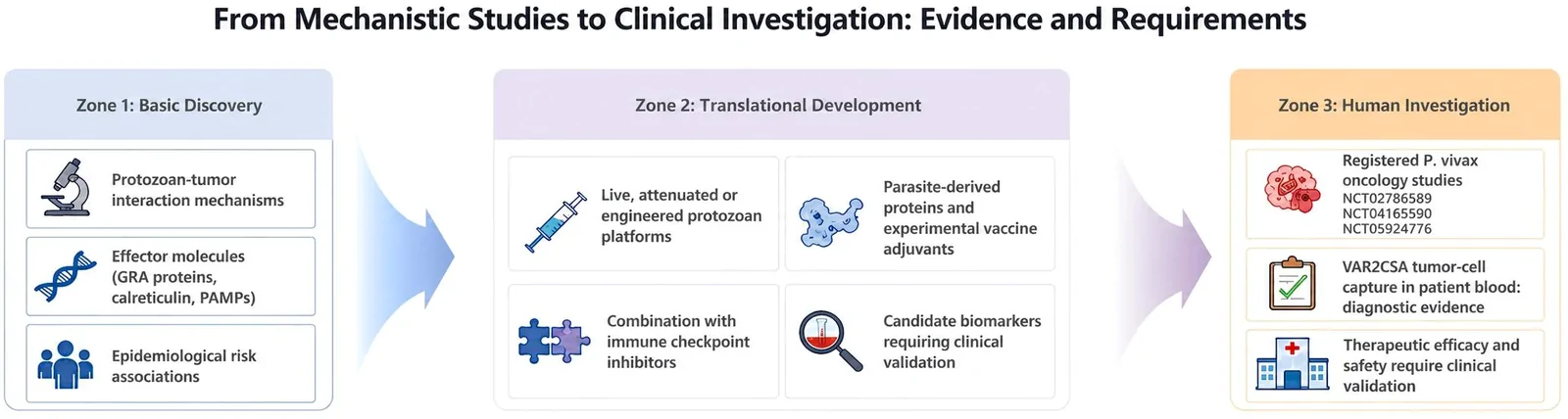
From mechanistic studies to clinical investigation: evidence and requirements. The three-zone layout separates discovery, translational development, and human investigation. Live, attenuated, engineered, and defined-product platforms require distinct safety and manufacturing evaluations. Registered P. vivax oncology studies (133–135) document investigational protocols, while VAR2CSA capture in patient blood documents diagnostic feasibility (143). Neither establishes therapeutic efficacy. Candidate biomarkers, persistence, clearance, and immune suppression require evaluation. Arrows indicate development requirements rather than completed clinical milestones or an inevitable progression to routine care.

### 1.1 Scope and approach to the narrative review

This article is a narrative synthesis, not a systematic review, scoping review, or meta-analysis. It integrates selected primary experiments, observational studies, and secondary reviews concerning protozoan infection, tumor immunity, and therapeutic development. Historical reports from the 1940s onward are included for context (6, 7, 10). The reference list is not an independently screened study cohort: multiple references may address the same mechanism, and reviews can overlap with the primary studies they summarize. Accordingly, reference counts are not used to estimate effect frequency or evidence strength. Tables provide qualitative assessments of study design, model relevance, and major limitations; no formal risk-of-bias score or exhaustive coverage is claimed. Targeted source verification during revision checked disputed claims against original publications and public trial registrations. The accompanying verification log records reference-level Europe PMC searches covering publication dates from 1 January 1945 to 14 September 2026, with Crossref used for a supplementary identifier check and IARC and ClinicalTrials.gov used for official records. These retrospective checks do not reconstruct the original literature-selection history and are distinct from a reproducible systematic literature search.

The principal scope is unicellular organisms commonly discussed as protozoa in medical parasitology, including Blastocystis, a stramenopile. Multicellular helminths are outside the main synthesis. Infections with Schistosoma haematobium, Opisthorchis viverrini, and Clonorchis sinensis have established carcinogenic classifications and are mentioned only for comparison (11, 12). Their exclusion is taxonomic and does not imply weaker evidence than the protozoan associations discussed here. The classification applies to the named species and should not be generalized to all schistosomiasis or all liver flukes.

### 1.2 Evidence categories and terminology

Four evidence categories are distinguished. Human observational studies describe associations and require attention to temporality, confounding, and reverse causation. Mechanistic studies identify cellular or molecular responses, sometimes outside a tumor model. Experimental interventions can support causality within a specified model, especially when pathway perturbation or rescue links a mechanism to a tumor endpoint; this does not establish causality in human cancer. Human therapeutic studies address safety or clinical outcomes, whereas trial registration documents a planned investigation rather than a result. These categories are complementary and are not a universal ranking: an observational human association and a mouse intervention answer different questions. **Tables 1**–**3** identify representative sources and limitations instead of assigning a species-wide net effect.

**Table 1:** Evidence profiles for selected protozoan tumor interactions.

Taxon	Evidence and examples	Tumor setting	Interpretation	Main limitation
Toxoplasma gondii	Cell and animal interventions (52, 56–58, 62–68)	Ovarian, melanoma, lung and other models	Tumor suppression in selected models; heterogeneous findings summarized in (9)	Construct and host dependence; human benefit unestablished
Plasmodium spp.	Rodent interventions (59, 71–76); human exposure assessment (8)	Lung, liver and breast models; endemic Burkitt lymphoma	Experimental suppression differs from P. falciparum malaria-associated cancer risk	Species, exposure and endpoints are not interchangeable
Trypanosoma cruzi	Protein, extract and antibody studies (53–55, 79–82)	Mammary, melanoma, colon and leukemia models	Vascular, direct-cell and immune-related effects	Selected preparations do not establish a benefit of natural infection
Trichomonas vaginalis	Human cohorts/meta-analyses (104, 105, 113, 116–121); cell assay (114)	Prostate outcomes and cervical neoplasia	Inconsistent human associations; supernatant inhibits cell growth *in vitro*	Different exposures/endpoints; no demonstrated human causal role
Cryptosporidium spp.	Susceptible mouse models (96–98, 110, 111); observational studies (126, 127)	Gastrointestinal and biliary lesions; human cancer populations	Experimental neoplasia in defined models; human associations	Severe immune deficiency limits extrapolation; temporal ambiguity in humans
Blastocystis spp.	Rat experiment (100); case-control and synthesis (101, 102, 109, 115)	Colorectal cancer	Exacerbation in a chemical model; unresolved human subtype associations	Small typing samples; culture selection, confounding and reverse causation
Leishmania spp.	Animal interventions and antigen delivery (84, 140)	Breast and HPV-associated models	Preclinical tumor-suppressive findings	Limited construct-specific evidence; no established oncology safety
Entamoeba histolytica	Infection mechanism review (43); clinical case series (131)	Opportunistic infection in cancer patients	Clinical susceptibility and infection biology	These sources do not establish a tumor-promoting or therapeutic effect
Neospora caninum	Exploratory animal intervention (83)	Experimental solid tumors	Preclinical tumor-suppressive activity	Limited model coverage; human benefit–risk unestablished

**Table 2:** Cellular targets and evidentiary limits of representative interventions.

Effector or strategy	Organism	Primary target	Observed evidence	Interpretive boundary
GRA15	T. gondii	Host innate STING signaling	Enhanced host defense during infection (15)	Infection mechanism; no tumor endpoint established by this citation
GRA16	T. gondii	Tumor-cell PP2A-B55/NF-κB	Suppressed lung cancer cell signaling and growth (52)	Direct cellular evidence; not patient efficacy
Attenuated or non-replicating constructs	T. gondii	Host antitumor immunity	Animal tumor suppression and checkpoint combinations (52, 56–58, 62–68)	Attenuation and replication control vary by construct
TcCRT	T. cruzi	Host endothelium; complement binding separately	Antiangiogenesis and experimental tumor inhibition (17, 54, 55, 79, 80)	Vascular effects are distinct from direct tumor-cell killing
Recombinant gp82	T. cruzi	Melanoma cells	Apoptosis in cell experiments (53)	Direct *in vitro* activity; systemic efficacy not inferred
VAR2CSA targeting ligand	P. falciparum	Oncofetal chondroitin sulfate	Cargo-targeting experiments and patient-sample cell capture (77, 142, 143)	Diagnostic capture is distinct from therapeutic benefit
Infection-associated host exosomes	Plasmodium models	Vascular signaling	Reduced angiogenesis in a lung cancer model (76)	Host-derived vesicles; not direct parasite-cell cytotoxicity
MDSC and Treg modulation	Plasmodium models	Host immune populations	Reduced suppressive-cell activity in mouse tumors (71)	Causal and pathway claims remain specific to that model
IL-12-stimulating protein	Eimeria	Dendritic cells	IL-12 release and animal antitumor activity (86)	Correlation with intestinal tumorigenicity is a distinct observation
Attenuated parasite or antigen vector	Leishmania	Host immune response	Animal intervention or HPV16 E7 delivery (84, 140)	Different constructs; no class-wide efficacy or safety claim

**Table 3:** Human observational evidence and limits of causal interpretation.

Exposure	Endpoint	Design and sources	Finding	Limitations
T. vaginalis seropositivity	Advanced or lethal prostate outcomes	Prospective/nested studies (117–121)	Positive findings in some studies; several null findings	Endpoint and cohort heterogeneity; causal role unestablished
T. vaginalis exposure	Prostate cancer occurrence	Cohorts and meta-analysis (113, 116, 117, 119, 120)	Inconsistent associations	Exposure misclassification and confounding; no validated risk estimate here
Blastocystis infection	Colorectal cancer	Case-control studies (101, 109, 125)	Prevalence or subtype associations vary	Small subtype samples, selection and reverse causation
Cryptosporidium infection	Cancer-patient infection or tissue detection	Meta-analysis/case report (126, 127)	Increased detection in some cancer settings	Colonization does not establish tumor initiation
Intestinal protozoan exposures	Colorectal cancer	Meta-analysis (122)	Pooled observational association	Heterogeneous organisms, detection and comparison groups
Mixed intestinal infections	Oncology populations	Surveys/case-control studies (128–132)	Opportunistic infection burden	Disease, treatment and exposure may increase susceptibility
E. histolytica	Infection in cancer patients	Case series (131)	Clinical infection and safety concern	Descriptive clinical evidence; no causal cancer inference

The endpoint is as important as the experimental design. Changes in cytokine concentration, immune-cell abundance, or gene expression describe a biological response, whereas tumor growth, metastatic burden, recurrence, and survival describe different aspects of cancer behavior. An increase in inflammatory cells is informative only when their location and function are considered alongside the tumor outcome. Likewise, fewer vessels may reflect an effect on endothelial cells, a consequence of reduced tumor size, or both. This review therefore evaluates the sequence from exposure to cellular response and then to a cancer endpoint. Where a study documents only one part of that sequence, the missing connection remains an explicit research question. This approach preserves the mechanistic value of infection studies while avoiding a premature assignment of therapeutic or carcinogenic meaning to every molecular change.

Tumor-promoting and tumor-suppressive describe experimentally observed effects on tumor growth, survival, or progression in the stated model. Antitumor immunity refers specifically to immune responses directed against tumors. Human observational findings are described as positive, null, or inconsistent associations rather than tumor-promoting effects. Direct tumor-cell effects, effects on host vasculature, and immune-mediated effects are identified separately. Shared pathway names do not establish equivalent activity across organisms.

## 2 Protozoan modulation of host immunity

Protozoa alter immune recognition and effector responses through species-specific strategies (13, 14). This section provides infection-immunology context. Unless a tumor endpoint is explicitly reported, the mechanisms described should be regarded as candidate links to cancer biology, not demonstrations of tumor suppression or promotion.

### 2.1 Innate recognition and evasion

Host pattern-recognition pathways detect protozoan components, while parasites can alter intracellular compartments and downstream signaling (13). T. gondii GRA15 enhances STING-dependent host defense in an infection model (15); that experiment alone does not establish an antitumor effect of GRA15. Type I and type II interferon responses have distinct, context-dependent roles in protozoan infection (16). T. cruzi calreticulin (TcCRT) binds complement components and inhibits classical complement activation (17), illustrating one of several complement-evasion strategies (18). Its vascular effects are considered separately in Section 3.4. Neutrophil extracellular traps can contribute to antiparasitic defense and tissue inflammation, but their tumor consequences require model-specific evidence (19).

Innate recognition provides a useful example of this distinction between detection and outcome. A host-defense signal may improve control of a parasite, alter the survival of an infected cell, or recruit additional immune populations. Each possibility can change the amount and location of subsequent exposure. Thus, the intensity of signaling measured after infection reflects both recognition and the parasite’s ability to persist. For GRA15, the experimentally established connection to STING supports a role in host defense; a cancer application would additionally require evidence that the same effector reaches a relevant cell population and changes tumor behavior through that pathway (15). Comparing an effector-deficient parasite with a complemented strain, while measuring parasite burden, would help distinguish loss of signaling from a secondary reduction in infection. This is particularly important when interpreting apparent differences between parasite strains that also differ in invasion, replication, or virulence.

Complement-related observations require a similarly precise interpretation. The ability of TcCRT to interact with complement components concerns survival at the host–parasite interface (17, 18). Endothelial responses to the same molecule address another biological compartment. These activities can motivate a multifunctional therapeutic hypothesis, but identifying several functions in one protein does not establish their order or relative contribution in a tumor. For a proposed mechanism, the relevant binding activity, responding cell, and downstream endpoint should be specified. This distinction guides experimental controls: a preparation that retains vascular activity but loses a complement interaction would answer a different question from complete removal of the protein. It also prevents complement evasion from being used as a general synonym for either immune activation or immune suppression in cancer.

### 2.2 Immune cell responses to infection

T. gondii can stimulate Th1 responses, and selected tumor models show reversal of immune suppression (20). Infection-induced conversion of NK cells to ILC1-like cells has also been demonstrated (21), but this finding does not by itself specify the effect on cancer. Plasmodium and Leishmania modify cytokine networks through distinct mechanisms (22). Antigen presentation, NK/ILC plasticity, and cytokine signaling overlap with processes important in tumor immunology (1–3); overlap provides a rationale for testing, not proof of a common tumor outcome.

The immune-cell findings are most informative when abundance is considered together with function. Dendritic cells can be present in a tumor without efficiently presenting tumor antigen, and an increase in infiltrating lymphocytes does not reveal whether they recognize tumor cells or parasite antigens (1–3). In the ovarian cancer experiments discussed below, restoration of antigen-presenting activity provides a more specific link to tumor immunity than a general increase in inflammation. The NK-to-ILC1-like conversion observed during Toxoplasma infection further illustrates why a cell’s phenotype and effector capacity cannot be inferred from its original lineage alone (21). For comparative studies, measuring antigen presentation, cytokine production, and tumor-directed cytotoxicity alongside cell counts would distinguish recruitment from functional reprogramming. The same principle applies to myeloid populations: a change in markers associated with activation should be interpreted with direct measurements of suppression or support of lymphocyte responses.

### 2.3 Epigenetic transcriptional and metabolic responses

T. gondii effectors modify host transcriptional and epigenetic programs (23), and infection can selectively alter translation of host transcripts (24, 25). Chromatin modulation is also described in other protozoan infections (26). These observations identify potential mechanisms of persistent host-cell alteration but do not establish that every affected program promotes cancer. MDSCs are important in tumor-associated immune suppression (4), while infection-related metabolic changes may affect the tumor microenvironment (27). The causal contribution of each process must be tested in the relevant parasite–tumor system.

Transcriptional, translational, and epigenetic measurements capture different layers of the host response. Increased transcript abundance need not produce a corresponding increase in protein, particularly when infection changes the translation of selected host messages (23–25). Moreover, a profile obtained from an infected tissue combines altered responses within cells with changes in the proportions of cell types present. A mechanistic account should therefore identify whether the signal arises in an infected cell, a neighboring uninfected cell, or an infiltrating immune population. This matters for therapeutic development because delivery of an isolated effector may reproduce only one component of the response to an intact organism. Time-resolved analyses and cell-specific perturbations would help distinguish a transient adaptation to infection from a sustained program that influences tumor growth. Persistent expression by itself remains insufficient to establish a stable cancer-associated phenotype.

### 2.4 Mucosal microbiota and extracellular communication

Mucosal protozoa interact with host barriers and resident microbiota. Tritrichomonas musculis activates the inflammasome and modifies resistance to mucosal infection (28); it also influences pulmonary immune responses and disease outcomes (29). These are infection and tissue-immunity findings, not evidence of a tumor switch. Microbiota composition and protozoan colonization can influence one another (30). Parasite communication, coinfection, extracellular vesicles, and microRNAs provide additional routes of host modulation (31–34). Their relevance to tumor behavior should be distinguished from their established roles in infection.

The mucosal setting adds a further layer of interaction because changes attributed to a protozoan may be mediated partly by the surrounding microbial community. The Tritrichomonas studies establish that intestinal colonization can modify local and distant immune responses, providing a rationale for examining communication between anatomical sites (28, 29). They do not identify a tumor-directed response. In a cancer experiment, useful comparisons would include the same parasite exposure under defined microbial conditions, with measurements of barrier integrity and community composition over time. Extracellular vesicles raise a related attribution problem: material recovered during infection can contain host-derived as well as parasite-derived components (33, 34). Describing its origin and purification is essential before assigning an effect to a parasite product. These distinctions allow microbiota and vesicle research to extend the mechanistic discussion without treating all infection-associated changes as direct actions on malignant cells.

### 2.5 Relevance to experimental cancer therapy

Some parasite-associated preparations inhibit cancer-cell growth *in vitro* (35), and a T. gondii-derived protein adjuvant has enhanced tumor-directed immunity in a murine colorectal cancer model (36). These findings motivate development of defined molecules and immune adjuvants, as discussed in reviews of parasite-derived interventions and shared immune-evasion pathways (37–41). Other infection settings can be harmful: persistent C. parvum infection changes tumor-associated transcriptional programs in mice (42), and Entamoeba histolytica modulates inflammatory NF-κB signaling (43). Veterinary and broader pathogen-associated cancer literature provide comparative context (5, 44), but neither establishes a common causal mechanism across the protozoa covered here.

The protein-adjuvant approach illustrates how a defined preparation can connect infection biology to a specific therapeutic question. In the murine colorectal cancer study, the parasite-derived protein was evaluated in the context of immunization with cancer cells, linking the intervention to a tumor-directed immune response (36). Its interpretation therefore differs from exposure of a cancer-cell line to a parasite extract. An adjuvant may improve recognition of an accompanying antigen even when it has little direct cytotoxic activity. Conversely, a molecule that reduces cell viability in culture may have no useful antigen-presenting function. Development should follow the activity actually demonstrated: antigen delivery and immune priming for an adjuvant, intracellular target engagement for an effector, or endothelial responses for a vascular agent. Treating these as separate starting points makes subsequent comparisons between platforms more informative.

## 3 Experimental tumor suppression and therapeutic investigation

Tumor-suppressive findings include early mouse experiments (6, 7) and more recent studies of attenuated organisms, recombinant proteins, and combination strategies (45–51). The following sections distinguish the cellular target of an intervention and the model in which an effect was observed. Preclinical efficacy is not treated as evidence of patient benefit.

### 3.1 Direct vascular and immune mediated mechanisms

Three mechanistic categories are useful. Direct tumor-cell effects include altered signaling or apoptosis after exposure to a defined effector or parasite product, as in GRA16 and gp82 experiments (52, 53). Vascular effects involve host endothelial cells and angiogenesis, as in TcCRT studies (54, 55); these are not direct tumor-cell cytotoxicity. Immune-mediated effects require host responses such as dendritic-cell activation, Th1 polarization, or tumor-reactive lymphocytes (56–59). A single intervention may engage more than one category. Cellular assays can isolate a mechanism but cannot establish its safety or efficacy in immunocompromised patients.

Recent reviews describe these overlapping mechanisms across experimental systems (47–51). However, recurrence of terms such as IFN-γ, macrophage polarization, or angiogenesis does not demonstrate mechanistic equivalence. Causal interpretation is strongest when a defined perturbation changes a tumor endpoint within the same model. **Table 2** therefore distinguishes representative intervention findings from infection-only mechanisms and identifies the supporting references.

For direct effects, the central questions are whether the molecule reaches malignant cells at an effective concentration and whether the response is selective relative to nonmalignant cells. For vascular effects, functional endothelial assays and tumor perfusion measurements address different parts of the mechanism. For immune-mediated effects, depletion or genetic disruption of the implicated population can test whether tumor control depends on that response. These approaches should be interpreted alongside controls for infection severity: a change in general health can influence tumor measurements without establishing tumor-selective activity. Rechallenge experiments add another dimension by asking whether protection persists after the initial intervention, although their interpretation depends on whether residual organisms or persistent antigen remain. Organizing experiments around these distinct questions avoids evaluating every candidate solely by the degree of short-term tumor-volume reduction.

### 3.2 Toxoplasma gondii

Intratumoral formalin-fixed T. gondii inhibited Lewis lung carcinoma growth in an early mouse study (10). Subsequent work using lysate antigen reported reduced CD31 expression (60), while live-infection experiments implicated Th1 responses and reduced angiogenesis in tumor suppression (61). These approaches differ in exposure, replication capacity, and mechanism and should not be considered interchangeable.

The comparison between fixed organisms, lysate, and live infection is particularly useful because it separates the presence of parasite material from the capacity for an ongoing host–parasite interaction. Formalin-fixed organisms supply a complex antigenic preparation, whereas a lysate changes the availability and arrangement of its components. Live or invasion-competent attenuated organisms can additionally deliver effectors into host cells. The early Lewis lung carcinoma and lysate studies consequently support different experimental possibilities rather than successive demonstrations of one identical treatment (10, 60, 61). Reduced CD31 expression provides a vascular observation, but identifying the responsible component would require further fractionation or pathway testing. The value of this earlier work is that it establishes several routes by which parasite-associated material can influence a tumor model, allowing later studies to ask which activities can be retained in a more defined preparation.

Avirulent or attenuated T. gondii reversed immune suppression in murine ovarian cancer and melanoma models, including protection against tumor rechallenge in the latter setting (56, 57). A non-replicating uracil-auxotroph model demonstrated a requirement for specific secreted parasite effectors in antitumor immunity (58, 62). Lactate-dehydrogenase-deficient and gamma-irradiated preparations have also been investigated (63–65). Reduced virulence or efficacy in mice does not establish complete replication control, absence of persistence, or safety in immunocompromised patients; these properties require evaluation for each construct.

Baird et al. provided a more detailed cellular explanation in the ovarian carcinoma model: the avirulent cps strain preferentially entered tumor-associated CD11c-positive antigen-presenting cells, and these cells regained cross-presentation and the ability to prime CD8-positive T-cell responses. The response was IL-12-dependent and MyD88-independent in that experimental setting (56). This links a change in the tumor immune compartment to a defined intervention rather than simply equating parasite-induced inflammation with tumor control. The melanoma rechallenge findings address a complementary issue, namely whether treatment can generate protection extending beyond the initially treated lesion (57). Together, these experiments support the investigation of local immune priming, while leaving open how tumor location, antigen availability, and prior immunity influence its reproducibility across cancer types.

The uracil-auxotroph experiments further separate replication from biological activity. Nonreplicating parasites can still interact with host cells, and Fox et al. showed that secreted rhoptry and dense-granule effectors contributed to the development of antitumor immunity in the ovarian tumor system (58). Thus, replication restriction does not mean that the preparation is immunologically inert. This is an attractive design principle because a candidate might retain selected cell-entry and signaling functions while limiting expansion. It also creates a characterization requirement: the relevant effector activity should be measured rather than inferred solely from viability or attenuation. Different strategies, including metabolic deficiency and irradiation, may preserve different combinations of invasion, secretion, and persistence. Their antitumor activity and host effects should therefore be assessed as properties of individual preparations, not transferred automatically from one attenuated strain to another.

Direct tumor-cell signaling has been investigated using GRA16, which increased PP2A-B55 and suppressed NF-κB activity in non-small-cell lung cancer cells (52). Protein-based approaches have also been studied in pancreatic cancer models (66). Immune-mediated combination experiments reported enhanced activity with anti-PD-1 treatment (67), while intratumoral type I ΔGRA17 parasites combined with PD-L1 blockade affected treated and distant tumors in mice (68). These findings support distinct, model-dependent mechanisms involving effectors and host immunity; the general role of IL-12/IFN-γ in immune activation does not establish that every Toxoplasma intervention uses the same pathway (20, 69).

GRA16 illustrates a distinct route from the whole-organism immune studies. In the lung carcinoma experiments, the PP2A-B55/AKT/NF-κB relationship was examined in tumor cells, providing a molecular account of a cellular response to the effector (52). This addresses intracellular signaling rather than restoration of dendritic-cell antigen presentation. The distinction has practical consequences: an effector-based product would need an effective delivery system and evidence of target engagement, whereas a locally administered organism may depend on interactions with several immune populations. Checkpoint combinations address yet another question. Improved control of treated and distant tumors after ΔGRA17 treatment with PD-L1 blockade supports testing systemic immune consequences of local intervention (68). Establishing whether the combination broadens tumor-specific immunity, extends its duration, or relieves a particular inhibitory pathway requires experiments designed around those alternatives.

### 3.3 Plasmodium species

Selected rodent tumor studies report Plasmodium-associated tumor suppression (70). In Lewis lung carcinoma, infection activated NK- and T-cell responses (59); other studies implicated changes in MDSCs, Tregs, and chemokine signaling (71). Liver cancer models reported altered macrophage function and angiogenesis (72), reduced HIF-1α-associated vascularization (73), and changes in recurrence or metastasis with EMT-related signaling (74). CD8+ T-cell responses contributed to inhibition of 4T1 breast tumors (75). Host exosomes obtained during infection inhibited angiogenesis in a Lewis lung carcinoma model (76), representing a host-derived vascular mechanism rather than direct parasite cytotoxicity. Separately, recombinant P. falciparum VAR2CSA targets oncofetal chondroitin sulfate for cargo delivery (77). These interventions must be distinguished from P. falciparum malaria in holoendemic populations and its association with endemic Burkitt lymphoma (8). Neither the positive animal findings nor a selected reference set supports a universally tumor-suppressive classification of Plasmodium.

The Lewis lung carcinoma work assessed both innate and adaptive responses, including NK-cell activity, cytokine production, and tumor-specific T-cell responses (59). These measurements help connect infection-associated immune stimulation with the observed tumor outcome. Subsequent reports on MDSCs and Tregs examine how the suppressive compartment changes during infection (71), while the liver and breast tumor studies extend the question to other anatomical and immunological settings (72–75). Comparing these systems should retain the identity of the rodent parasite, tumor model, exposure route, and timing. A similar decrease in tumor growth can arise through different combinations of immune recruitment and vascular change. Conversely, differences between models may reflect the accessibility of the tumor to a response rather than an intrinsic difference in the potency of the organism.

The host-exosome and VAR2CSA studies make the separation of platforms especially clear. Exosomes recovered during infection carry information about the infected host state; their vascular activity is therefore not equivalent to showing that a parasite directly kills an endothelial or tumor cell (76). Recombinant VAR2CSA instead uses a parasite protein’s binding properties to recognize a tumor-associated glycan and deliver a selected cargo (77). The latter strategy does not require reproducing malaria as the therapeutic exposure. These examples suggest different experimental priorities: identification of active vesicle components for the former, and target distribution, binding specificity, and cargo behavior for the latter. Their inclusion under Plasmodium reflects molecular origin, not a single shared clinical intervention. This distinction also resolves the apparent tension between antitumor laboratory studies and malaria-associated cancer risk in endemic populations.

### 3.4 Trypanosoma cruzi

T. cruzi preparations and proteins have been studied through several mechanisms (78). TcCRT inhibited angiogenesis in chick chorioallantoic membrane assays (54) and altered endothelial-cell proliferation, migration, and vessel formation, with tumor-growth inhibition in a murine mammary model (55). Its complement-binding activity and vascular effects are distinct observations; the cited evidence does not justify attributing all antitumor activity to a single complement–VEGF mechanism (17, 79, 80). Recombinant gp82 produced apoptosis in melanoma cells, supporting a direct cellular effect (53). In contrast, parasite extracts elicited protective immune responses in chemically induced colon and mammary cancer models (81), and antibody cross-reactivity with leukemia cells represents an additional immune-related line of investigation (82). These findings concern defined preparations and models, not a beneficial effect of natural Chagas disease in patients.

TcCRT is supported by a sequence of vascular assays that is more informative than tumor size alone. The reported inhibition of endothelial proliferation, migration, and capillary-like organization, together with ex vivo and *in vivo* vascular observations, provides a rationale for examining angiogenesis as a contributor to tumor control (54, 55). Nevertheless, the extent to which this activity explains an individual tumor response depends on delivery and the tumor’s vascular requirements. Antibody cross-reactivity and responses to parasite extracts pose different mechanistic questions: recognition of a tumor-associated target must be distinguished from recognition of shared or broadly distributed cellular components (81, 82). Similarly, gp82-associated apoptosis in cultured melanoma cells establishes a direct cellular observation but does not supply the pharmacological information needed for systemic administration (53). The T. cruzi literature is therefore best read as several candidate mechanisms emerging from different preparations, each requiring its own route of validation.

### 3.5 Additional protozoan models

Exploratory studies report tumor-suppressive activity of Neospora caninum (83), live-attenuated Leishmania preparations in breast cancer models (84), and Babesia microti infection with macrophage activation in murine melanoma (85). An Eimeria-derived protein stimulated dendritic-cell IL-12 release and showed activity in animal tumor experiments (86). The associated intestinal tumorigenicity observations should not be treated as a controlled demonstration that Eimeria infection prevents human cancer. These studies expand the range of candidate interventions, but neither a shared mechanism nor clinical safety has been established across these organisms.

These additional organisms broaden the experimental landscape without providing a meaningful basis for ranking species by efficacy. Whole-organism treatment, an attenuated preparation, and a defined Eimeria-derived protein differ in exposure as well as in their proposed immune targets (83–86). A positive finding in one tumor type may justify replication in another model, but comparisons between unrelated studies cannot identify the most effective platform. The useful next step is to determine which response is necessary in each system and whether it persists under a second set of tumor conditions. Reporting organism identity, preparation, route, dose, and treatment timing alongside the immune and tumor endpoints would allow the smaller literatures to contribute to the same mechanistic framework as the more extensively studied Toxoplasma and Plasmodium models.

### 3.6 Microbiota and checkpoint combinations

Research on intratumoral microbes and gut microbiota provides a broader context for immune modulation and treatment response (87–90). Direct evidence for individual protozoa should be separated from findings in bacteria or fungi. Protozoan–microbiota interactions (30) and viruses of protozoan parasites (91) may be relevant to future studies, but the latter literature does not establish a cancer treatment based on viruses targeting protozoa. Defined microbiota and checkpoint-combination experiments are needed to test these proposed interactions.

For a checkpoint combination, the mechanistic question is whether the protozoan intervention changes a process that limits the response to blockade. Increased antigen availability, improved antigen presentation, altered myeloid suppression, and recruitment of effector cells are distinct possibilities. A study that compares the combination with each single treatment can establish incremental activity, but the magnitude and nature of the interaction require an appropriate design. Microbiota composition may also differ between animal facilities or treatment groups, making it a potential modifier rather than an established explanation for every combination effect (30, 87–90). Measuring microbial context together with tumor-specific immune function would help determine whether an observed improvement arises from the protozoan preparation, a community change, or both. Such analyses are more useful than extending general findings about the tumor microbiome directly to a particular protozoan.

The tumor-suppressive literature encompasses direct signaling effects, vascular effects, and host immunity. A single pathway cannot account for all interventions. Conversely, reports from chronic infection do not demonstrate that each beneficial intervention necessarily becomes tumor-promoting with time. The next section distinguishes experimental cancer-associated effects from observational human associations.

## 4 Chronic infection and cancer associated findings

Persistent antigen exposure, inflammatory injury, and altered tissue signaling offer plausible links between infection and cancer (92–94). Their contribution differs across parasites and host conditions. The evidence below includes infection-induced lesions in susceptible animals, exacerbation of chemically induced carcinogenesis, and observational associations in humans; these outcomes should not be combined into a universal causal sequence.

### 4.1 Candidate inflammatory and immune pathways

T-cell exhaustion involves sustained inhibitory-receptor expression and loss of effector function under persistent stimulation (92). Chronic inflammation can also influence epithelial signaling and the tumor microenvironment (93, 94). These general mechanisms motivate protozoan-specific experiments but do not demonstrate an NF-κB/STAT3–MDSC/Treg–cancer pathway in every organism. Likewise, metabolic and leptin-related interactions described in parasitic infection are contextual hypotheses unless directly linked to a tumor endpoint (95).

The proposed inflammatory pathways involve several transitions that should be examined separately. Persistent stimulation can alter immune-cell function; inflammatory injury can change the epithelial environment; and an established tumor can itself recruit suppressive cells and sustain inflammation (92–94). Because these processes can occur together, their co-occurrence does not establish which came first. An experimentally useful distinction is between initiation of a new lesion, promotion of an already initiated lesion, and changes in the growth of an established cancer. Histological endpoints, timing of exposure, and the presence of another carcinogenic stimulus determine which question a model can answer. Measurements of oxidative stress or inflammatory signaling add biological plausibility, but linking them to neoplasia requires a corresponding change in lesion development when the proposed pathway is perturbed. This preserves the value of the mechanism while making the causal claim proportional to the experiment.

### 4.2 Cryptosporidium and experimental gastrointestinal neoplasia

C. parvum infection has been associated with gastrointestinal neoplasia in severely immunodeficient mouse models (96, 97). Experimental work reported ileo-cecal adenocarcinoma with changes in Wnt-associated signaling and epithelial junctions (98), and transcriptional studies identified tumor-microenvironment-related responses (42). These experiments support pathogenic effects under the tested conditions. They do not establish human carcinogenesis or prove that every accompanying molecular change is necessary for tumor formation. Reviews propose inflammatory, immune, and signaling mechanisms (99), which should be interpreted alongside the immunodeficiency and treatment conditions of the underlying models.

The Cryptosporidium models are important because they move beyond detecting an organism in a tumor-bearing host to observing neoplastic lesions after experimental exposure. Their interpretation nevertheless depends on the permissive host environment. Severe immunodeficiency changes both control of parasite replication and the tissue response to prolonged infection (96–98). Consequently, the model addresses what infection can do under those conditions rather than estimating what occurs after a typical exposure in an immunocompetent person. The observations involving Wnt-associated signaling, epithelial junctions, and cytoskeletal organization suggest candidate connections between infection and altered epithelial architecture (98). Demonstrating that any one connection is required would involve preserving comparable infection while selectively modifying the host response. This distinction between an experimentally induced lesion and a fully established molecular pathway is central to evaluating the strength of the evidence.

### 4.3 Blastocystis and colorectal cancer

Blastocystis exacerbated colon carcinogenesis in a chemically induced rat model (100). This supports an effect on progression in that experimental setting, not independent initiation of cancer by infection. Human studies report associations, including an ST1 signal in a Saudi case-control study (101), while pooled observational evidence remains heterogeneous (102). Proposed mechanisms include inflammatory signaling, oxidative stress, barrier changes, and dysbiosis (103). Their occurrence and causal contribution vary by model and have not established a human cancer-causing role. Subtype findings and methodological limitations are examined in Section 5.3.

The chemically induced rat model offers a different type of evidence from the Cryptosporidium studies. Because carcinogenesis was initiated by another exposure, a greater tumor burden following Blastocystis exposure is most directly interpreted as modification of that experimental process (100). It does not answer whether the organism can initiate a tumor in otherwise comparable animals without the initiating treatment. Human case-control studies address a further question: whether carriage is more common in people who already have cancer (101, 102). These designs can be complementary when their endpoints are kept distinct. A coherent mechanistic program would connect the observed association with specific host responses and then test those responses in models that distinguish initiation from promotion. At present, subtype findings and proposed barrier or microbiota mechanisms provide hypotheses for that program rather than a complete causal account of human colorectal cancer.

### 4.4 Trichomonas vaginalis and reproductive tract outcomes

Meta-analyses report associations between T. vaginalis infection and cervical or other reproductive-tract neoplasia (104, 105). Infection detection, cervical inflammation, intraepithelial lesions, and invasive cancer are distinct endpoints. A pooled association across such outcomes is not proof that T. vaginalis initiates invasive cervical cancer. HPV coinfection, sexual behavior, screening, and exposure measurement can influence the observed relationships. Mucosal inflammation and interactions with other pathogens are candidate explanations, not established causal mechanisms shared with intestinal protozoa. The prostate cancer literature is considered separately in Section 6.1.

Combining reproductive-tract outcomes can obscure where an association arises. Infection may be measured at the time of an abnormal screening result, in a biopsy-defined lesion, or after diagnosis of invasive disease; each setting has different opportunities for selection and detection bias. HPV status is especially important because it may be related both to the cancer endpoint and to the characteristics of the population in which T. vaginalis is detected (104, 105). A more informative synthesis separates infection ascertainment, lesion grade, and invasive cancer, and then considers whether the association remains after relevant adjustment. This does not dismiss a possible contribution from inflammation. It identifies the additional evidence needed to establish whether T. vaginalis has an independent effect, modifies another infection’s consequences, or is primarily a marker of shared exposure and clinical detection.

### 4.5 Inflammatory bowel disease and dysplasia

An observational study examined protozoan infection and dysplasia in patients with ulcerative colitis (106). Concurrent infection and dysplasia do not establish temporal progression or exclude disease severity and treatment as alternative explanations. Broader parasitology reviews discuss suspected cancer associations beyond the established trematode carcinogens (11, 107); helminth findings are comparative context and are not counted as protozoan evidence.

In ulcerative colitis, the underlying inflammatory disease provides an additional explanation for the coexistence of infection and dysplasia. Disease activity, mucosal damage, surveillance intensity, and immunomodulatory treatment should be considered when evaluating the reported association (106). The relevant comparison is not simply infected versus uninfected tissue at one visit, but the sequence of inflammation, treatment, infection detection, and subsequent histological change. Repeated sampling could help distinguish persistent exposure from a transient finding, while comparable surveillance would reduce differences in the opportunity to detect dysplasia. This setting illustrates a broader point: cancer-associated inflammation can be both a proposed mediator of infection effects and a pre-existing condition that changes susceptibility to infection. An observational study must address these competing explanations before the inflammatory pathway can be assigned a causal direction.

Collectively, these studies support further testing of infection-related inflammation and epithelial injury. They do not demonstrate that chronic infection invariably promotes cancer, or that an acute-to-chronic transition explains every difference between tumor-suppressive experiments and human associations.

## 5 Candidate modifiers of protozoan tumor interactions

Strain or subtype, exposure burden and duration, tissue tropism, host immune status, and observation timing may help explain heterogeneous findings. Evidence that a factor modifies outcome within an experiment must be separated from an explanation inferred by comparing unrelated studies. A reversal of tumor outcome should only be described as demonstrated when comparable conditions have been tested directly.

### 5.1 Experimental heterogeneity in toxoplasma studies

The systematic review of T. gondii experiments identified both tumor-suppressive and tumor-promoting observations (9). Substantial heterogeneity prevented quantitative meta-analysis. This supports a descriptive framework organized around model and exposure, but does not provide a pooled estimate or establish a universally validated switch controlled by strain, duration, or immune status.

A context-dependent account becomes useful when it generates comparisons that could distinguish competing explanations. For example, two strains tested in different tumor models cannot isolate a strain effect, and an early intervention cannot be directly compared with a late intervention if the organism dose also changes. The Toxoplasma synthesis draws attention to such heterogeneity, but its descriptive findings do not estimate independent contributions of each variable (9). A stronger design would hold the tumor and host background constant while varying one exposure characteristic, followed by a prespecified test of interactions between characteristics. The endpoint should also remain consistent: reduced growth of an established tumor and prevention of tumor establishment are not interchangeable demonstrations of the same effect. This reframes the proposed switch as a set of experimentally separable questions rather than a single transition inferred from the literature as a whole.

### 5.2 Context dependent IFN-γ signaling

IFN-γ can enhance antigen presentation and cytotoxic immune responses while also inducing adaptive resistance, including PD-L1 and IDO1 expression in relevant tumor contexts (108). Direction depends on the responding cell, tumor type, signaling duration, concentration, and interaction with other pathways. A general rule that acute or low-intensity exposure suppresses tumors whereas chronic or high-intensity exposure promotes them is not established across protozoan models. The dose-and-timing hypothesis is a testable explanation for selected observations and requires experiments that measure exposure and perturb IFN-γ signaling within the same system.

Cellular location provides one way to organize the apparently divergent consequences of IFN-γ. A signal that improves antigen presentation or supports effector function can coexist with a tumor-cell response that increases inhibitory signaling (108). Measuring a circulating concentration alone cannot determine the balance of these responses within a lesion. Similarly, an association between persistent signaling and poor tumor control does not show that reducing all IFN-γ activity would improve the outcome. A discriminating experiment would measure the pathway in the relevant cell populations and examine how selective disruption affects both parasite control and tumor behavior. Changes in parasite burden must be included because they can alter the stimulus itself. The resulting evidence could support a model-specific schedule or combination strategy, but the optimal direction of intervention should emerge from those experiments rather than from an assumed acute-beneficial/chronic-harmful rule.

### 5.3 Strain subtype and tissue specificity

The Saudi study of Mohamed et al. enrolled 74 colorectal cancer patients, 64 patients with other cancers, and 80 non-cancer controls. It used PCR-RFLP and seven pairs of subtype-specific STS primers, rather than a panel restricted to ST1/ST2/ST5; these were the subtypes recovered, not the full assay capability (101). Ali et al. enrolled 100 colorectal cancer patients and 100 controls in Egypt, but the reported sequence-based subtype comparison contained only 10 isolates per group. ST7 occurred in three case isolates and no control isolates; overall culture positivity was 52% versus 42%, a nonsignificant difference (P = 0.101) (109). Geographic variation, culture selection, typing methods, and small subtype samples may contribute to the different findings, but their relative contributions were not tested. These results do not establish ST1 or ST7 as a validated cancer-risk marker or demonstrate subtype-dependent reversal of tumor behavior. A human-derived C. parvum isolate produced gastrointestinal and biliary neoplasia in susceptible mice (110), illustrating model-specific pathogenic potential rather than proving tissue tropism as an independent switch variable.

The two Blastocystis subtype reports should therefore be read as population-specific signals with different sampling constraints. The number of people enrolled is not the number of successfully typed isolates, and the latter determines how much information is available about subtype distribution (101, 109). A finding in three isolates can motivate replication but yields an unstable basis for assigning risk to an entire subtype. Culture-based selection may also change the subset that proceeds to sequencing, making direct comparison with other typing approaches difficult. The appropriate follow-up is a larger, consistently sampled study that reports the full path from stool collection through positivity, amplification, and subtype assignment. Concurrent measurement of mixed infection, organism burden, and relevant host characteristics would help determine whether a subtype association persists independently. The present data support neither a universal ST1 pattern nor a competing universal ST7 pattern.

### 5.4 Infection burden duration and host cost

C. parvum lesion severity correlated with infection intensity in SCID mice (111). This finding concerns a defined susceptible model and should not be generalized to a human dose–cancer relationship. Chronic T. gondii infection caused sustained cachexia and dysbiosis in mice (112), demonstrating host costs rather than a measured reversal of tumor suppression. Burden and persistence are therefore relevant to safety as well as efficacy. Attenuated and non-replicating strategies require separate validation of clearance, tissue distribution, and antitumor activity (58, 63, 64).

Duration and burden also need separate definitions. The inoculated dose records the initial experimental exposure, whereas the subsequent tissue burden reflects replication, clearance, and distribution. Time since inoculation is not necessarily equivalent to continuous exposure at the same intensity. The relationship between infection intensity and lesions in susceptible Cryptosporidium models makes this distinction biologically relevant (111). For a therapeutic experiment, serial measurements could relate the duration of a useful immune response to the persistence of viable organisms. Host cost should be evaluated on the same timeline: tumor reduction accompanied by sustained weight loss or tissue injury represents a different outcome from tumor control with recovery of host function. The chronic Toxoplasma cachexia observations reinforce the need to measure these consequences independently rather than use tumor size as a complete indicator of benefit (112).

### 5.5 Trichomonas findings across different experimental questions

The heterogeneous association between T. vaginalis exposure and prostate cancer risk (113) is not directly contradicted by inhibition of established cancer cells *in vitro*. Zhu et al. tested parasite culture supernatant, not live-parasite coculture, and reported reduced proliferation and increased apoptosis in PC-3 and DU145 cells (114). Population studies concern infection and disease occurrence or prognosis; cell assays concern an isolated product exposure and short-term cell behavior. Differences in exposure, endpoint, and biological context prevent these findings from demonstrating a switch within one system. The same distinction applies when interpreting Blastocystis experiments alongside unresolved human associations (115).

The culture-supernatant result identifies a tractable product-discovery question: which component of the preparation produced the cellular response, and at what exposure relative to nonmalignant cells (114)? Answering it would require fractionation, appropriate medium controls, and confirmation that the activity is reproducible across preparations. That work could proceed even if prospective studies ultimately found no association between natural infection and prostate cancer. Conversely, a population association would not identify the active material in a supernatant experiment. The two literatures become genuinely connected only when a defined exposure and mechanism can be related across models. Describing them as different experimental questions preserves their respective contributions and avoids treating an apparently opposite result as evidence for a reversal that neither study directly tested.

The five proposed modifiers organize hypotheses rather than form a predictive equation. Experimental evidence for specific effects is available in some Toxoplasma and Cryptosporidium models, whereas the Blastocystis subtype evidence discussed here is observational and small. Most combinations of strain, burden, tissue, host state, and timing have not been tested factorially. Future studies should distinguish a true interaction from differences in study design and should report null as well as positive results.

## 6 Population evidence, opportunistic infection, and safety

Human studies address infection exposure, cancer occurrence, prognosis, and susceptibility to opportunistic disease. These questions are distinct from efficacy in animal models. In particular, cancer and its treatment may increase infection susceptibility, creating an association without infection having initiated the cancer.

### 6.1 Trichomonas vaginalis and prostate cancer

Some prospective serological studies reported positive associations with prostate cancer or particular aggressive outcomes (116, 117). In contrast, Tsang et al. did not find an association between T. vaginalis serostatus and mortality among men with prostate cancer (118). Studies of a multiethnic cohort and of advanced disease also reported null findings (119, 120), and elevated cytokines or seropositivity at diagnosis were not associated with higher Gleason grade or lethal disease in another study (121). Differences in exposure ascertainment, screening, confounding, and endpoint definitions complicate comparison. These data do not establish T. vaginalis as a causal prostate cancer risk factor, nor do they support describing the advanced or lethal disease evidence as consistently positive.

The prostate literature also illustrates why incidence and prognosis should be synthesized separately. A study of subsequent diagnosis asks whether prior exposure predicts cancer occurrence, whereas follow-up after diagnosis asks whether exposure predicts progression or death among men who already have cancer (116–121). Selection into the latter group changes the population under observation. Serological exposure measurements also do not directly specify the timing, persistence, or anatomical location of infection. Differences in screening can influence the detection of indolent disease, and differing definitions of aggressive or advanced cancer can alter which outcomes are compared. A convincing synthesis should therefore present the positive and null findings according to endpoint and exposure measurement, rather than count them as votes for or against a single causal claim. The coexistence of positive prospective associations and null mortality findings identifies uncertainty about particular relationships; it does not establish a uniform effect across the course of prostate cancer.

### 6.2 Colorectal cancer associations and reverse causation

Meta-analytic and cross-sectional evidence reports intestinal protozoan infections among colorectal cancer patients (122, 123). Case-control work also examines Blastocystis alongside other intestinal organisms (124). Sulzyc-Bielicka et al. found increased Blastocystis prevalence before oncological treatment and interpreted the observation in terms of opportunistic infection; pretreatment detection does not establish that infection preceded cancer (125). Cryptosporidium meta-analyses likewise cannot determine causal direction from prevalence comparisons (126). Detection of C. meleagridis in the tissue of an immunocompetent patient with colon cancer is evidence of colonization, not proof of tumor initiation (127).

Pretreatment sampling removes one important ambiguity, namely exposure to anticancer therapy before the specimen is obtained, but it leaves several others. The tumor may already have altered local tissue, nutrition, bowel function, or healthcare contact by the time infection is measured (125). Detection at diagnosis therefore cannot establish the sequence needed for an initiation hypothesis. Stool positivity and demonstration of organisms in tissue likewise answer different questions, with tissue detection providing localization rather than a history of exposure (127). Prospective samples collected before diagnosis would strengthen temporal interpretation, especially if paired with repeated measurements and comparable detection in controls. Existing prevalence studies remain useful for describing the clinical population and developing candidate mechanisms. Their value is greatest when presented as evidence about coexistence and susceptibility, with causal direction left for designs capable of resolving it.

### 6.3 Opportunistic infection in oncology populations

Studies in Jordan, Iran, and Malaysia describe intestinal protozoan infection in cancer patients, with differences in detection methods, comparison groups, and microbial context (128–130). Case reports and series document clinically consequential infections, including E. histolytica (131). A study of hematological malignancies also reported intestinal protozoan infection and exposure-related factors (132). These studies inform infection surveillance and clinical vulnerability; they do not demonstrate that the detected organisms caused the underlying malignancies.

For oncology populations, the immediate significance of these reports lies in the infection itself. A clinically consequential infection can complicate the interpretation of symptoms or host inflammatory measurements even when it has no established role in causing the malignancy (128–132). The same issue affects translational research: a candidate designed to stimulate immunity must be evaluated in hosts whose treatment or disease may impair control of the organism. Natural infection cohorts cannot directly establish the safety of an engineered therapeutic strain, but they identify relevant forms of vulnerability that should inform preclinical models and eligibility criteria. Separating infection surveillance from carcinogenesis also improves reporting. Studies should state whether their purpose is to detect opportunistic disease, estimate exposure prevalence, or test a cancer-risk hypothesis, because each purpose requires a different comparator and timeline.

Prospective exposure measurement, consistent endpoints, and adjustment for confounding are needed to assess causal hypotheses. Opportunistic infection should be analyzed separately from cancer initiation. Heterogeneity among population studies can motivate subtype and burden analyses, but does not itself validate the five-variable framework.

## 7 Translational development and registered clinical investigation

Protozoan-based interventions include live organisms, attenuated or engineered strains, purified proteins, and host-derived products. These approaches have different mechanisms, manufacturing requirements, and safety concerns. Clinical maturity must be assessed separately for each platform.

Human investigation is documented by registered Plasmodium immunotherapy studies, including NCT02786589 in advanced non-small-cell lung cancer, NCT04165590 in advanced malignant solid tumors, and NCT05924776 in advanced ovarian cancer (133–135), with records accessed 14 September 2026. These records concern blood-stage P. vivax exposure and should not be conflated with non-replicating engineered vectors or recombinant proteins. Registration documents study intent; it does not establish recruitment completion, favorable outcomes, or a clinically acceptable benefit–risk balance. The lung cancer record had no posted results at the accessed record. The combinations and engineered platforms summarized below are supported principally by preclinical studies, with separate human-sample diagnostic work for VAR2CSA. Neither evidence type establishes therapeutic efficacy in patients.

### 7.1 Combinations with chemotherapy and radiotherapy

Plasmodium immunotherapy combined with gemcitabine inhibited tumor growth and metastasis in murine Lewis lung carcinoma, with changes in EMT-associated signaling (136). A separate preclinical study investigated combination with radiotherapy (137). These findings provide a rationale for testing interactions with established treatments; they do not establish clinical synergy or superiority to standard care. Mechanistic similarity to other animal combinations, including Toxoplasma with PD-L1 blockade (68), remains an inference unless the relevant pathways are tested in comparable systems.

Combination studies should distinguish a greater response from evidence of a specific interaction between treatments. A regimen can outperform either component alone without establishing that the mechanisms are synergistic. The schedules, doses, and endpoints of the gemcitabine and radiotherapy experiments should therefore be interpreted within their individual designs (136, 137). Scheduling is biologically relevant because an intervention intended to prime immune cells may be influenced by treatments that alter those same populations. This suggests testing several sequences rather than assuming that simultaneous administration is optimal. Tumor control should be evaluated alongside infection burden and host toxicity so that an apparent gain does not conceal a narrower tolerable exposure range. Mechanistic assays can then ask whether the combination changes antigen release, immune priming, vascular function, or another specified process, providing a basis for selecting a reproducible regimen.

### 7.2 Antigen delivery and vaccine platforms

Engineered protozoan platforms have been studied as antigen carriers. Attenuated Plasmodium sporozoites expressing MAGE-A3 induced antigen-specific responses in a murine lung cancer model (138), and a glypican-3 delivery strategy was investigated for hepatocellular carcinoma (139). Recombinant Leishmania tarentolae expressing HPV16 E7 (140) and engineered T. cruzi vectors (141) provide additional animal proof of concept. Antigen-specific immune responses do not establish patient efficacy, and the safety of one parasite construct cannot be transferred to another.

Antigen-carrier experiments separate the capacity to stimulate immunity from the specificity of the response being stimulated. The engineered MAGE-A3, glypican-3, HPV16 E7, and T. cruzi vector studies use different antigen–platform combinations and should be interpreted accordingly (138–141). A response against the vector may coexist with a response against the encoded antigen, so the relevant assays need to distinguish them. Important comparisons include the carrier without the target antigen and the antigen delivered through another preparation. Tumor antigen expression also matters when judging whether an immune response can act on the chosen cancer model. These considerations make platform engineering a problem of matching delivery, antigen presentation, and tumor recognition. They further explain why a strong immune response to a protozoan vector, by itself, is insufficient evidence that the vector will improve control of an antigenically different tumor.

### 7.3 VAR2CSA targeting and human sample diagnostics

Recombinant VAR2CSA binds oncofetal chondroitin sulfate and has been investigated for tumor targeting (77). The glycosaminoglycan participates in integrin signaling and cell motility (142), but targeting selectivity and off-target effects require evaluation for each cargo. VAR2CSA also captured circulating tumor cells from human patient blood independently of EpCAM (143). This is human-sample diagnostic evidence, distinct from administration of a therapeutic product or demonstration of improved clinical outcomes. It therefore should not be classified simply as a murine therapeutic experiment.

The distinction between targeting and treatment is especially important for VAR2CSA. Binding to a tumor-associated structure establishes a means of recognition, but the behavior of a therapeutic construct also depends on its attached cargo, tissue exposure, and release or internalization (77, 142). A diagnostic capture assay instead tests recovery of cells from a specimen under controlled conditions. The EpCAM-independent patient-blood work demonstrates that this approach can identify tumor cells through an alternative binding strategy, extending the biological observation into a human-sample application (143). It does not test systemic tolerability or therapeutic delivery. For diagnostic development, the next questions concern analytical reproducibility and clinical interpretation of captured cells; for treatment, they concern distribution, target engagement, and toxicity. Keeping these paths separate allows the human-sample evidence to be recognized without overstating the clinical maturity of a drug platform.

### 7.4 Combination with a tumor antigen

Aguilar-Guzmán et al. investigated human survivin and TcCRT together in a murine melanoma setting (144). This is a preclinical antigen/protein combination, not evidence of efficacy with an approved survivin-targeted drug. The observation motivates mechanistic evaluation of antigen-directed immunity alongside TcCRT-associated effects (54, 55, 79, 80).

The survivin–TcCRT combination should be interpreted at the level of the tested antigen and protein preparation (144). Combining a tumor-associated antigen with a parasite-derived molecule could alter immune recognition, vascular responses, or both, but identifying their relative contributions requires appropriate single-component and mechanistic controls. An improved tumor outcome does not establish that TcCRT will combine similarly with every treatment directed at the same tumor-associated target. The distinction is relevant when discussing translation because a vaccine-like antigen preparation and a pharmacological inhibitor have different mechanisms and exposure requirements. The study supplies a rationale for further development of the particular combination, while the broader value of pairing antigen-directed immunity with vascular modulation remains a hypothesis to be tested across additional models.

Translation requires platform-specific evidence. Live parasites need validated attenuation or replication control, monitoring of persistence and shedding, tissue-distribution studies, and an effective rescue or clearance strategy. Recombinant products require identity, purity, potency, immunogenicity, and biodistribution assessment. Oncology studies must consider immune suppression, organ function, prior exposure, treatment interactions, and prespecified stopping criteria. Manufacturing consistency and regulatory review are development requirements, not evidence that any candidate is ready for routine clinical use. Registered studies should be evaluated through reported safety and outcome data rather than their existence alone.

For live or invasion-competent products, attenuation, persistence, and clearance are separate development questions. Attenuation describes reduced pathogenicity under tested conditions; persistence concerns how long and where the organism remains; clearance concerns whether it can be reliably eliminated. A candidate that performs well in immunocompetent animals may behave differently under the immunosuppressive conditions relevant to cancer treatment. Evaluation should therefore include both the intended therapeutic exposure and conditions that could reveal loss of control. Pre-existing immunity may also change distribution or the response to repeated administration, so it should be measured rather than assumed to improve either safety or efficacy. An effective rescue strategy would need evidence that it reaches the relevant anatomical sites and controls the actual therapeutic construct. These are platform-specific requirements, particularly when genetic modification changes the organism’s susceptibility or tissue behavior.

Manufacturing and clinical evaluation should be connected to the proposed mechanism. For an immune adjuvant, a potency assay should reflect its ability to support the relevant immune response; for a targeting ligand, binding and cargo-related activity are distinct attributes. Batch consistency in these functions is more informative than a concentration or viability measure alone. Early human investigation should then separate feasibility, dose-limiting toxicity, biological activity, and tumor response, with a clear plan for attributing adverse events to infection, inflammation, or concomitant therapy. Patient selection should be justified by the product’s mechanism and the host conditions studied during development, rather than by a general designation of an immunologically inactive tumor. These steps provide a way to translate mechanistic findings into testable clinical questions while preserving the distinction between an investigational protocol and demonstrated patient benefit.

## 8 Limitations of the current evidence base

Most mechanistic and tumor-suppression experiments use cell lines or murine models, often transplantable tumors, laboratory strains, and artificial inoculation routes. These designs can reveal causal mechanisms within the tested setting but may not reproduce natural exposure, spontaneous human tumors, or treatment-related immune suppression. Strain, dose, timing, and host background vary, and direct comparative experiments are limited. Mechanisms established during infection without a tumor endpoint require additional validation before being assigned a cancer effect.

Model choice can strengthen a mechanistic inference while narrowing its applicability. A simplified cell system isolates a molecular response but omits immune and vascular interactions. A transplantable tumor permits controlled comparisons but may differ from a tumor developing in its native tissue over a prolonged period. Immunodeficient models enable some questions while changing the host processes central to others. These differences should guide triangulation rather than be treated as interchangeable replications. Reproducing an effect across models with deliberately different features can identify which parts of the mechanism are robust and which depend on the experimental setting. Within each model, randomization, blinded endpoint assessment, prespecified analyses, and complete reporting of exclusions would improve interpretability. The absence of these details from an accessible report should be recorded as uncertainty, not replaced by an assumption that they were performed.

Human observational evidence is vulnerable to confounding, selection bias, exposure misclassification, and reverse causation. Cancer, treatment, and healthcare contact can alter both infection susceptibility and detection. HPV coinfection and sexual or screening-related factors are particularly relevant to reproductive-tract studies. Longitudinal evidence is limited for many organism–cancer pairs. Selective publication and citation of positive experiments may also distort the apparent balance of findings; the narrative selection used here cannot quantify this bias.

Statistical significance alone is also an incomplete basis for comparing this literature. A small study with a large apparent effect and a larger study with a more modest estimate may differ in precision, exposure measurement, or population composition. Pooling can summarize available estimates but cannot repair inconsistent endpoint definitions or establish temporal ordering absent from the contributing designs. Negative and inconclusive results are therefore essential to evaluating reproducibility, particularly in small subtype analyses and exploratory animal studies. Future syntheses would benefit from clearly separated datasets for human incidence, prognosis, opportunistic infection, and experimental treatment. Such separation would permit focused questions about bias and comparability before any quantitative analysis, rather than producing a single summary measure from biologically different relationships.

Translation presents additional uncertainty. Attenuation may be incomplete, and uncontrolled replication, immunopathology, off-target tissue involvement, and pre-existing immunity can alter benefit and risk. Immunocompromised patients may respond differently from the animals used to establish proof of concept. Although registered Plasmodium studies specify investigational protocols (133–135), their existence does not establish a generally validated patient-selection strategy or class-wide safety. Candidate-specific dose margins, persistence, manufacturing, and regulatory requirements remain essential. Section 7 separates these development questions from clinical outcomes and diagnostic sample studies.

This narrative review does not use exhaustive study selection, formal risk-of-bias scoring, or quantitative synthesis. Its proposed contextual framework is therefore an organizing hypothesis. It should guide explicit comparisons and falsifiable experiments rather than be interpreted as a validated predictor of whether infection will benefit or harm a patient.

## 9 Conclusion and outlook

Protozoan–tumor research spans cellular mechanisms, animal interventions, human associations, and early translational investigation. These evidence categories support different conclusions and should remain distinct when assessing causality and therapeutic relevance.

Tumor-suppressive and cancer-associated findings coexist across the literature. Shared signaling pathways provide mechanistic candidates, but the direction and consequence of their activity depend on the tested system. Infection-related GRA15–STING signaling, tumor-cell GRA16 effects, and context-dependent IFN-γ responses are not interchangeable demonstrations of one mechanism (15, 52, 108).

Strain or subtype, burden and duration, tissue tropism, host status, and observation timing are useful variables for study design. Their joint predictive value remains unvalidated. The Toxoplasma synthesis (9), small Blastocystis subtype comparisons (101, 109), and differences between Trichomonas epidemiology and culture-supernatant experiments (113, 114) motivate further testing rather than establish a universal switch.

Attenuated organisms, defined molecules, and antigen carriers show preclinical potential (55–58, 77, 138–141, 144). Registered Plasmodium studies document human investigation (133–135), while VAR2CSA has also been examined using patient samples for diagnostic capture (143). Neither registration nor diagnostic feasibility substitutes for evidence of therapeutic efficacy and safety.

Priorities include standardized strain and subtype reporting, measurement of exposure and persistence, comparisons across tumor models, and prospective human studies with clearer temporal ordering. Candidate biomarkers such as interferon activity or myeloid-cell states require predictive validation. For therapeutic development, attenuation, clearance, tissue distribution, and immune-mediated toxicity should be tested alongside efficacy, particularly under conditions resembling oncology-related immune suppression. Host costs, including cachexia and dysbiosis observed during chronic Toxoplasma infection, must also be considered (112).

A practical research sequence is to begin with a reproducible effect in a defined system, identify the responding cell and necessary pathway, and then examine whether the relationship survives changes in exposure and host context. Candidate markers should be evaluated prospectively for their ability to predict outcome before treatment, rather than selected only because they change after a response has occurred. Human association studies and therapeutic development can inform one another, but their validation criteria remain distinct: temporal and confounding control for an etiological hypothesis, and product-specific benefit and harm for an intervention. This approach would retain the breadth of protozoan biology while producing narrower conclusions that can be independently tested. It would also make unsuccessful replication informative by showing which proposed contextual explanation does or does not account for the result.

A context-dependent approach can clarify heterogeneous findings without assigning a fixed beneficial or harmful identity to a protozoan species. Clinical development should proceed from reproducible, platform-specific mechanisms and demonstrable safety rather than from a general expectation that infection will improve tumor immunity.
